# Metabolic and transcriptomic profiling during wheat seed development under progressive drought conditions

**DOI:** 10.1038/s41598-023-42093-2

**Published:** 2023-09-11

**Authors:** Ryosuke Mega, June-Sik Kim, Hiroyuki Tanaka, Takayoshi Ishii, Fumitaka Abe, Masanori Okamoto

**Affiliations:** 1 Graduate School of Sciences and Technology for Innovation, Yamaguchi University, Yamaguchi, 753-8515 Japan; 2 RIKEN Center for Sustainable Resource Science, Yokohama, 230-0045 Japan; 3 Institute of Plant Science and Resources, Okayama University, Kurashiki, 710-0046 Japan; 4 Faculty of Agriculture, Tottori University, Tottori, 680-8553 Japan; 5 Arid Land Research Center, Tottori University, Tottori, 680-0001 Japan; 6 Division of Basic Research, Institute of Crop Science, National Agriculture and Food Research Organization (NARO), Tsukuba, 305-8518 Japan; 7 Center for Bioscience Research and Education, Utsunomiya University, Utsunomiya, 321-8505 Japan

**Keywords:** Plant physiology, Plant stress responses, Drought, Seed development

## Abstract

Globally, bread wheat (*Triticum aestivum*) is one of the most important staple foods; when exposed to drought, wheat yields decline. Although much research has been performed to generate higher yield wheat cultivars, there have been few studies on improving end-product quality under drought stress, even though wheat is processed into flour to produce so many foods, such as bread, noodles, pancakes, cakes, and cookies. Recently, wheat cultivation has been affected by severe drought caused by global climate change. In previous studies, seed shrinkage was observed in wheat exposed to continuous drought stress during seed development. In this study, we investigated how progressive drought stress affected seed development by metabolomic and transcriptomic analyses. Metabolite profiling revealed the drought-sensitive line reduced accumulation of proline and sugar compared with the water-saving, drought-tolerant transgenic line overexpressing the abscisic acid receptor TaPYL4 under drought conditions in spikelets with developing seeds. Meanwhile, the expressions of genes involved in translation, starch biosynthesis, and proline and arginine biosynthesis was downregulated in the drought-sensitive line. These findings suggest that seed shrinkage, exemplifying a deficiency in endosperm, arose from the hindered biosynthesis of crucial components including seed storage proteins, starch, amino acids, and sugars, ultimately leading to their inadequate accumulation within spikelets. Water-saving drought tolerant traits of wheat would aid in supporting seed formation under drought conditions.

## Introduction

Bread wheat (*Triticum aestivum* L.) is one of the most widely cultivated and consumed crops globally. Many approaches to develop higher-yield wheat cultivars have been attempted. However, studies on flour quality are relatively rare compared with those on yield improvement, even though flour quality is also important because most wheat grain is processed into wheat flour to produce bread, noodles, pancakes, cakes, and cookies. The qualities of these products are determined by three features: grain protein content, grain starch content, and grain hardness^1^.

In particular, the elasticity of wheat dough is dependent on the content and composition of seed storage proteins (SSPs), including gluten, which confers its unique viscoelastic property on wheat dough products^2,3^. Gluten consists of two types of proteins, glutenin and gliadin. Glutenin is subdivided into high molecular weight glutenin subunits (HMW-GSs) and low molecular weight glutenin subunits (LMW-GSs); both types contribute to dough elasticity and resistance to stretching. Gliadin is associated with dough extensibility and viscosity. Starch is another factor that determines flour quality, and wheat flour is 70–80% starch^4^. Starch is subdivided into amylose and amylopectin. Amylose comprises short, straight chains of glucose molecules; amylopectin comprises long, branched glucose chains. The ratio of these two types of starch molecules determines noodle quality^5,6^. Grain hardness is used to evaluate market grade and greatly influences milling and baking quality. This trait is determined mainly by the SSP puroindoline. The most important genes for grain texture were found at the Hardness (Ha-D) locus, including *puroindoline a* (*Pina-D1*), *puroindoline b* (*Pinb-D1*), and *GRAIN SOFTNESS PROTEIN-1* (*GSP-1*)^7,8^.

These main components are biosynthesized in the endosperm during seed development to accumulate as nutrients for the next generation’s growth. During this developmental period, drought stress affects the final yield and the important components that contribute to good flour quality. Under drought conditions, glutenin and gliadin contents change, starch and arabinoxylan (a dietary fiber) accumulation is inhibited, and grain hardness increases slightly^9–12^. The composition of seed components can affect flour quality because of abiotic stresses, such as heat, drought, and their combination^13–15^. In a previous study, we showed that free amino acid and monosaccharides increased instead of polysaccharides, such as starch^11^. This suggested that polymer formation from amino acids and sugars was probably inhibited by drought stress. Because these polymers are important components of wheat flour, environmental-stress-tolerant wheat genotypes are needed to ensure constant end-use quality. However, little is known about how biosynthesis of these main components is affected by drought stress during seed development.

Therefore, it would be useful to understand the mechanism by which wheat overcomes drought stress during seed development to understand determinants of flour quality. Though many papers have reported that seed morphology is inhibited by drought stress^16,17^, little is known about how wheat plants cope with drought stress during seed maturation. Thus, we conducted multi-omics analyses combining transcriptome and metabolome analyses to obtain a comprehensive understanding about the effects of drought stress on flour quality using a transgenic, drought-tolerant wheat line overexpressing wheat abscisic acid receptor 4 (TaPYL4) named TaPYLox. TaPYLox exhibited a transcriptome pattern like wheat plants exposed to mild drought stress, even under control conditions, because of its high ABA sensitivity^11^. During drought stress, TaPYLox reduced its water consumption and sustained seed quality. Thus, water-saving TaPYLox is expected to aid the biosynthesis of SSPs and starch in seeds by providing a constant water supply to seeds under drought conditions. We performed various analyses on metabolites such as starch and SSPs and the transcriptome of TaPYLox to compare its characteristics with the non-transgenic control genotype. We found characteristic differences in gene expression and metabolites involved in the biosynthesis of SSPs and starch.

## Results

### Starch granules and seed storage proteins in seeds exposed to drought

**Figure 1:**
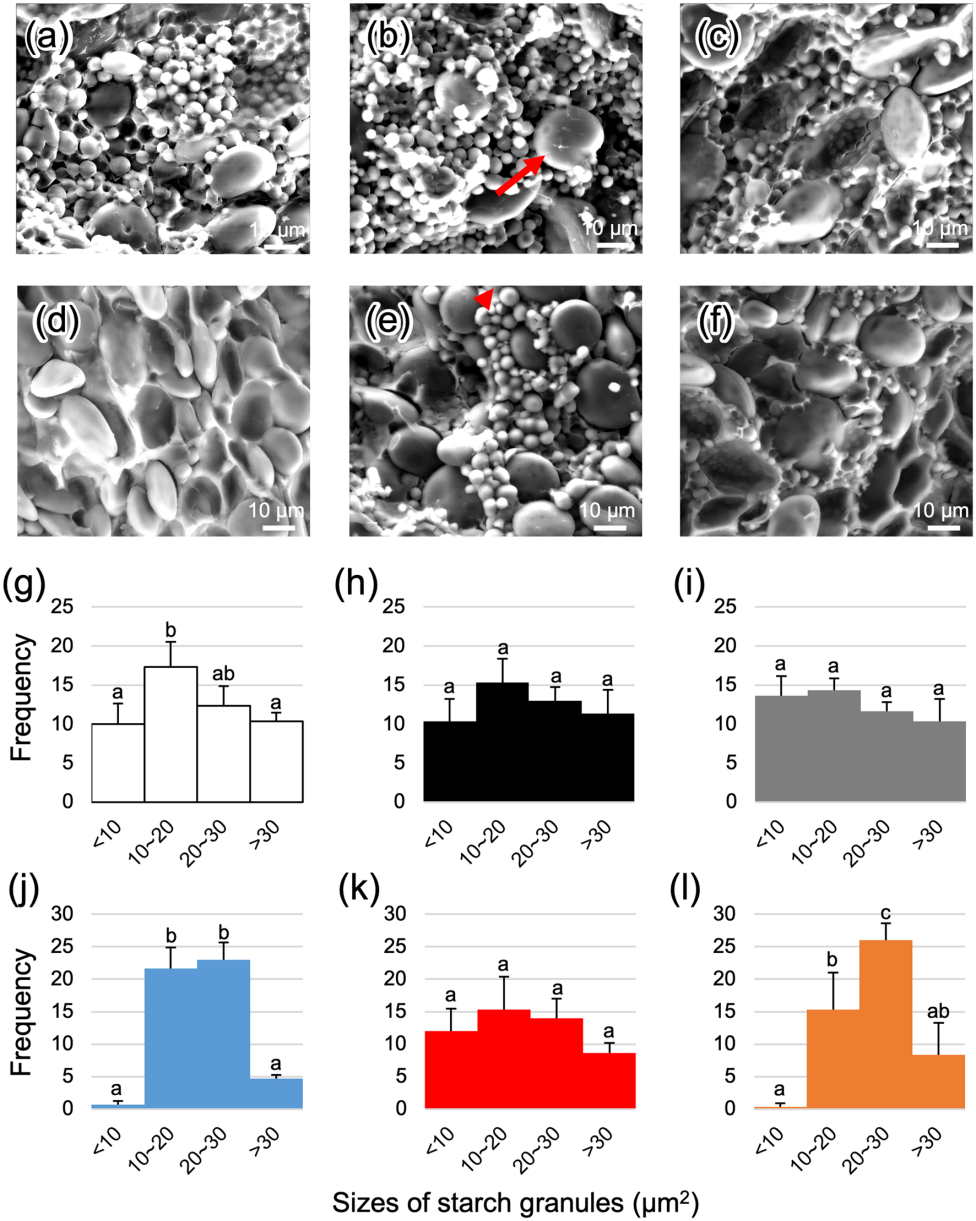
Starch granules in dry seeds cultivated under WTR and DR. (**a**–**f**) Cross-sectioned seeds were photographed under a SEM. The red arrow and arrowhead indicate starch granule A and starch granule B or the initial state of A. (**g**–**l**) Sizes of starch granules (µm^2^) were measured using Image J software (https://imagej.nih.gov/ij/download.html). (**a** and **g**) CW, (**b** and **h**) L8W, (**c** and **i**) L17W, (**d** and **j**) CD, (**e** and **k**) L8D, and (**f** and **l**) L17D. Different letters denote significant differences (P < 0.05) based on the Tukey–Kramer test.

Cross-sections of dry seeds of TaPYLox genotypes L8, L17, and C, which is the null segregant of TaPYLox, were observed by SEM. Starch granule size was measured using Image J based on the photos (Fig. 1a–f), and size-dependent histograms were drawn. Under the well-watered condition (WTR), the composition of starch granules did not largely differ between the three lines. However, there was only a different trend that 10–20 µm^2^ ranged granule in C was significantly higher than those in L8 and L17 (Fig. 1g–i). This suggests that increased ABA sensitivity may have some effects on starch granule development. Whereas, the composition of starch granules in C changed more than that of L8 under the drought condition with a limited water-supply (DR), and L17 showed a change intermediate between those of C and L8. According to Fig. 1j–l, the minimum- and maximum-sized granules decreased in numbers. Instead, intermediate-sized starch granules were increased in stressed seeds. The number of > 30 µm^2^ granules in C was lower than that in L17. There were two types of starch granules, designated A and B. The A granule was large, and the B granule was small, although in the initial stages of seed development, A granule could not be distinguished from B. This suggests that de novo formation of B granules halts prior to A granule growth by drought stress.

**Figure 2:**
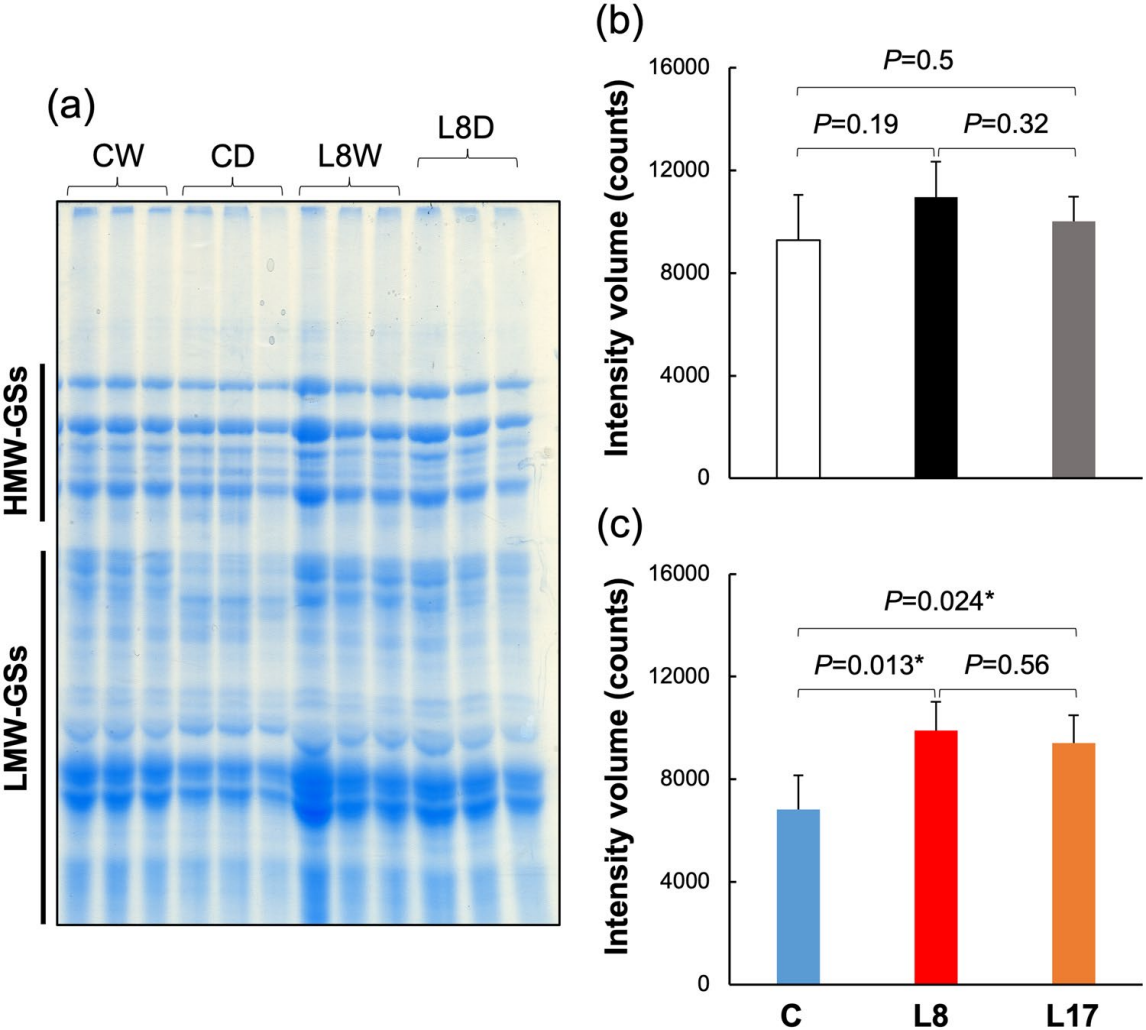
Amounts of glutenin in dry seeds cultivated under WTR and DR. (**a**) SDS–PAGE gel photo. The intensity, based on band volume, was measured using the specific bands for glutenin on the SDS–PAGE gel. The full-size gel photo is shown in Fig. S9. Protein samples from seeds grown under WTR (**b**) and DR (**c**) were loaded on the gel. Student’s *t* tests were conducted by using the “ggpubr” package of the R program^35^.

We performed SDS–PAGE using a protein solution including glutenin. The results are shown in Fig. 2. The amounts of glutenin were calculated by scanning the density of each band. The amounts of glutenin did not differ between C, L8, and L17 under WTR but declined in the seeds of C cultivated under DR.

### Transcriptome analysis of spikelets under progressive drought conditions

**Figure 3:**
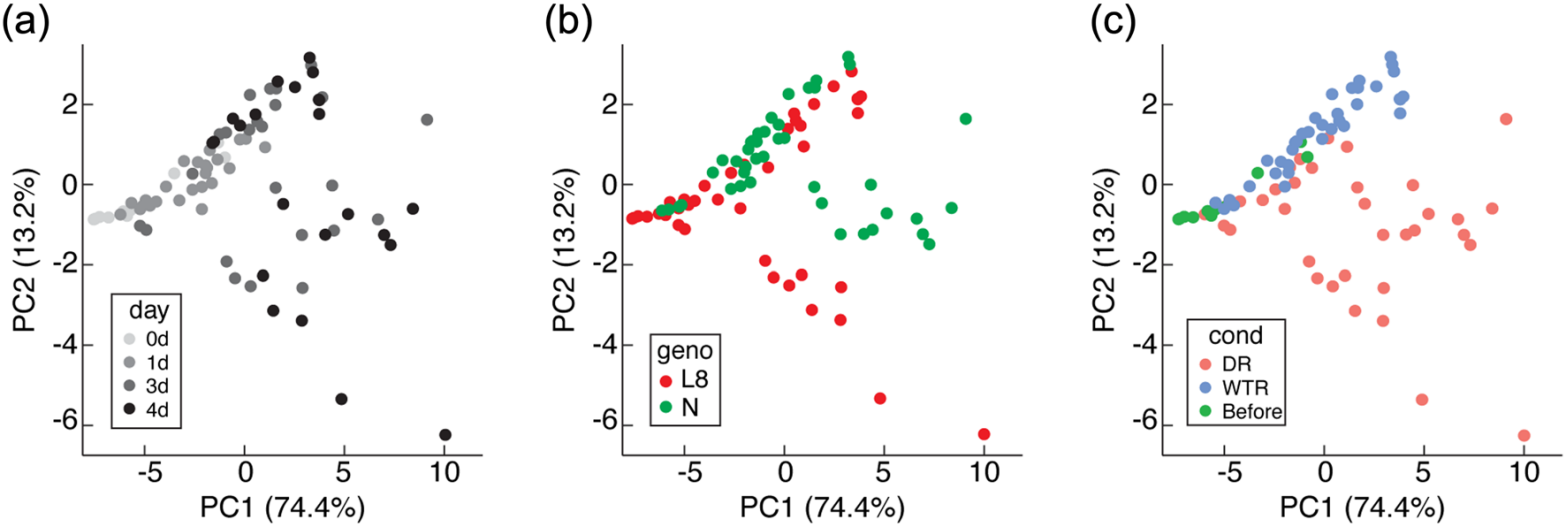
Principal component analysis (PCA) of the transcriptome. PCA score plot of developing wheat seeds at days 2, 7, and 9. Samples were collected from different DR and WTR conditions according to their transcript profiles. (**a**) Treatment time trend, (**b**) genotype trend, and (**c**) conditions trend.

**Figure 4:**
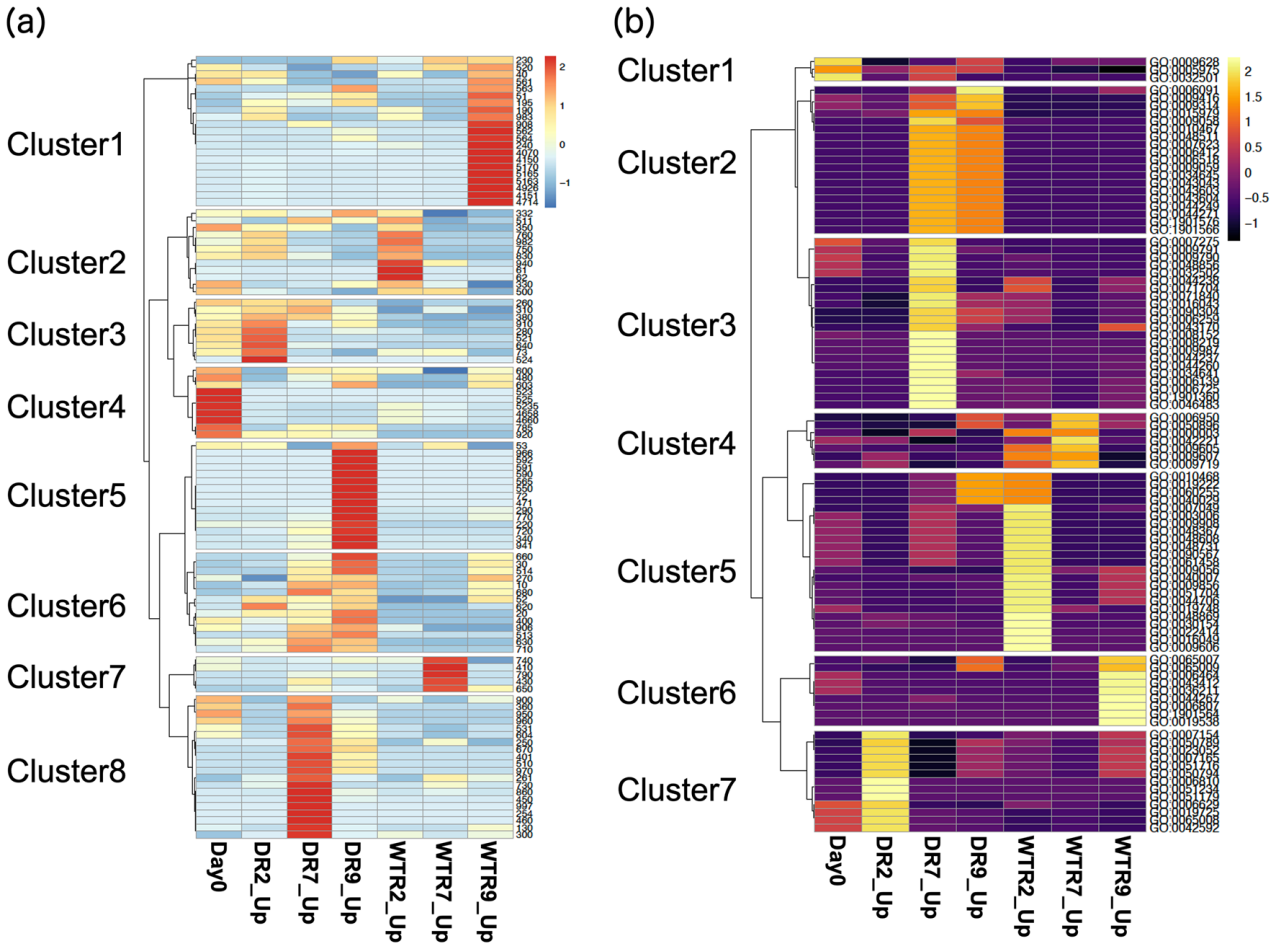
Hierarchical cluster analysis of the transcriptome. (**a**) KEGG pathway numbers, including DEGs, used for drawing the heatmap. (**b**) GO numbers, including DEGs, used for drawing the heatmap.

**Figure 5:**
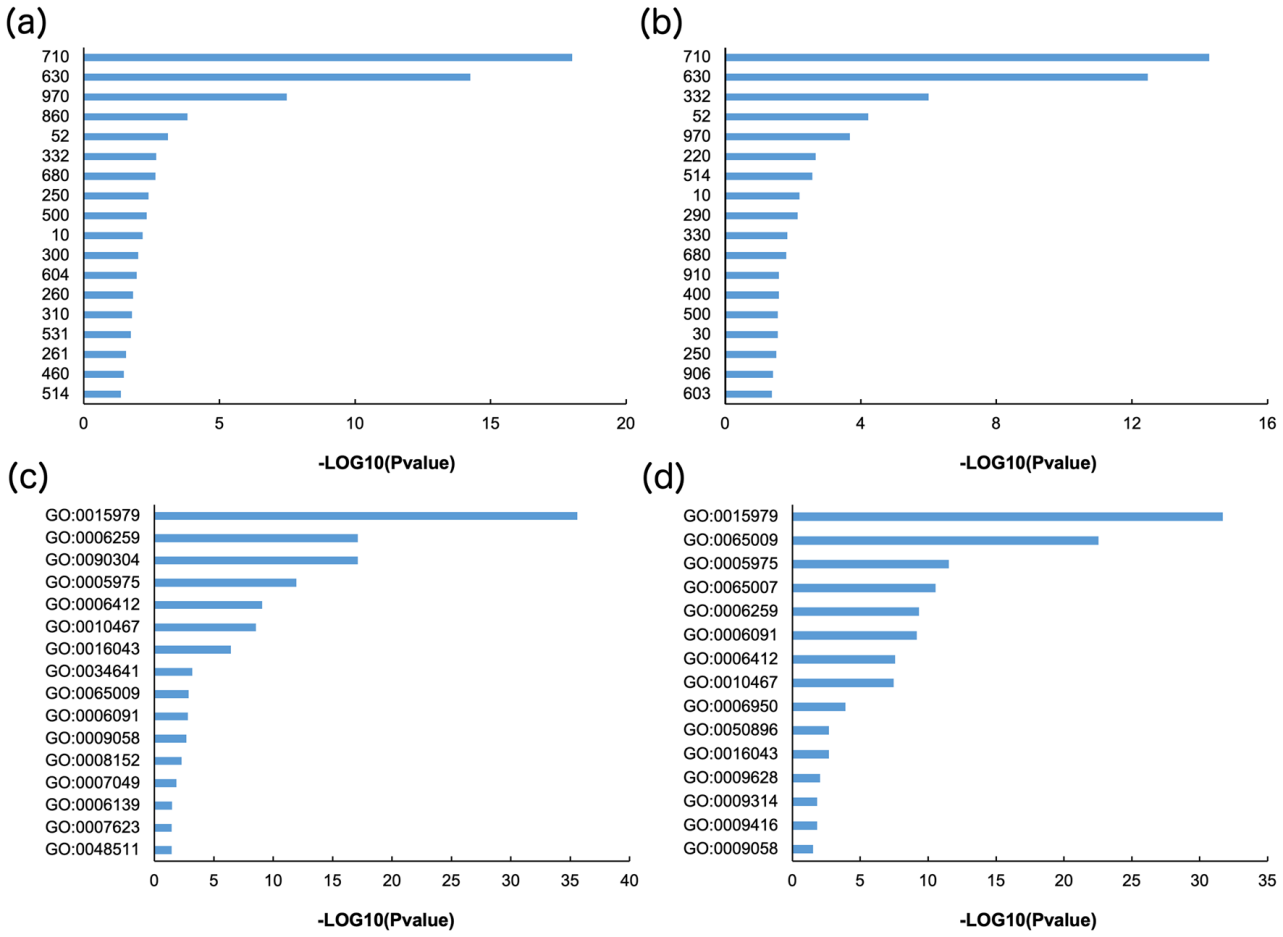
KEGG and GO pathway enrichment analyses of the transcriptome. Enrichment analyses were performed based on KEGG pathway and GO numbers at days 7 (**a** and **c**) and 9 (**b** and **d**).

Transcriptome analysis was performed to validate the gene expression underlying the mechanism determining seed quality using spikelet tissues harvested before drought (0) and at days 2, 7, and 9 in plants under WTR and those under DR (Fig. S1). Two-dimensional PCAs show that the trends in the presence and absence of responses to drought and the trends of genotypes under drought conditions were observed in the directions of the x- (PC1) and y-axes (PC2), respectively (Fig. 3). Interestingly, the transcriptome of drought-tolerant L8 changed more than that of drought-sensitive C. Hierarchical clustering analysis was then performed to evaluate differences in the effects of drought stress between C and L8 using KEGG pathway analysis and the Gene Ontology (GO) database (Fig. 4). The KEGG pathway analysis indicated clusters 5, 6, and 8 were notable because many genes upregulated in L8 were found because of their roles in drought stress (Fig. 4a). In the GO analysis, clusters 2 and 3 were notable (Fig. 4b). Most of the GOs in cluster 2 belonged to GO:0006412 (GO term, translation). Likewise, most of GOs in cluster 3 belonged to GO:0006259 (DNA metabolic process). These results suggest that translation-related gene expressions in L8 were higher than in C at DR days 7 and 9. At DR day 7, DNA metabolic processes changed remarkably. Interpretations of the KEGG and GO numbers shown in Fig. 4 are summarized in Table S1. Considering clusters observed at DR days 7 and 9, GO enrichment analyses with DEGs (differentially expressed genes,  > twofold change and P < 0.05) during DR on these days were performed in KEGG and GO (Fig. 5). The GO terms were selected in GO slim (https://www.ebi.ac.uk/QuickGO/help/slims). As a result, the KEGG analysis indicated there were genes involved in photosynthesis-related pathways (710, 860, 630 and 260), aminoacyl-tRNA biosynthesis (970), starch metabolism-related pathways (500, 52, and 10) at DR days 7 and 9. Genes involved in Arg and Pro biosynthesis (220 and 330, respectively) were also observed. In GO, GO:0006412 and GO:0006259 were commonly observed (Fig. 4b). Besides, the percentages of DEGs belonging to protein metabolic process (GO:0019538) within all upregulated ones under each condition were higher than those of carbohydrate metabolic process (GO:0005975) particularly at DR7 and 9 in Table S2. This suggests that protein metabolism tends to fluctuate rather than carbohydrate metabolism by severe drought stress.

Comprehensively evaluating the KEGG pathway and GO results, translation and starch metabolism appeared to affect the results observed in Figs. 1 and 2. Actually, DEGs of SSPs were not observed at DR days 7 and 9 (Table S3). The gene IDs of SSPs were characterized recently^18^. According to the characterization, there were four HMW-GSs, 17 low LMW-GSs, and 65 gliadin-forming genes in bread wheat. At DR day 7, only one LMW-GS and 16 gliadin genes were downregulated, and only three gliadin genes were upregulated. At DR day 9, three gliadin genes were downregulated, and only one LMW-GS and 23 gliadin genes were upregulated. These results suggest that glutenin gene expression contributed little to the maintenance of L8 glutenin levels under drought conditions. Therefore, glutenin biosynthesis could be more dependent on differences in translational activity between C and L8 than transcriptional activity.

In addition to SSPs, the other two components, starch and grain texture, were included as determinants. According to Gao et al.^18^, 45 starch biosynthetic genes were characterized. As shown in Table S3, one up- and one down-regulated DEG were observed out of 45 at DR day 7. These results suggest that maintenance of transcription in L8 could affect starch biosynthesis. Of the puroindoline genes involved in grain texture, one and nine DEGs were up and downregulated, respectively, at DR day 7. At DR day 9, seven and one DEGs were up- and downregulated, respectively. Unfortunately, there were no discernible trends in these results.

### Metabolomic analysis of spikelets under progressive drought conditions

**Figure 6:**
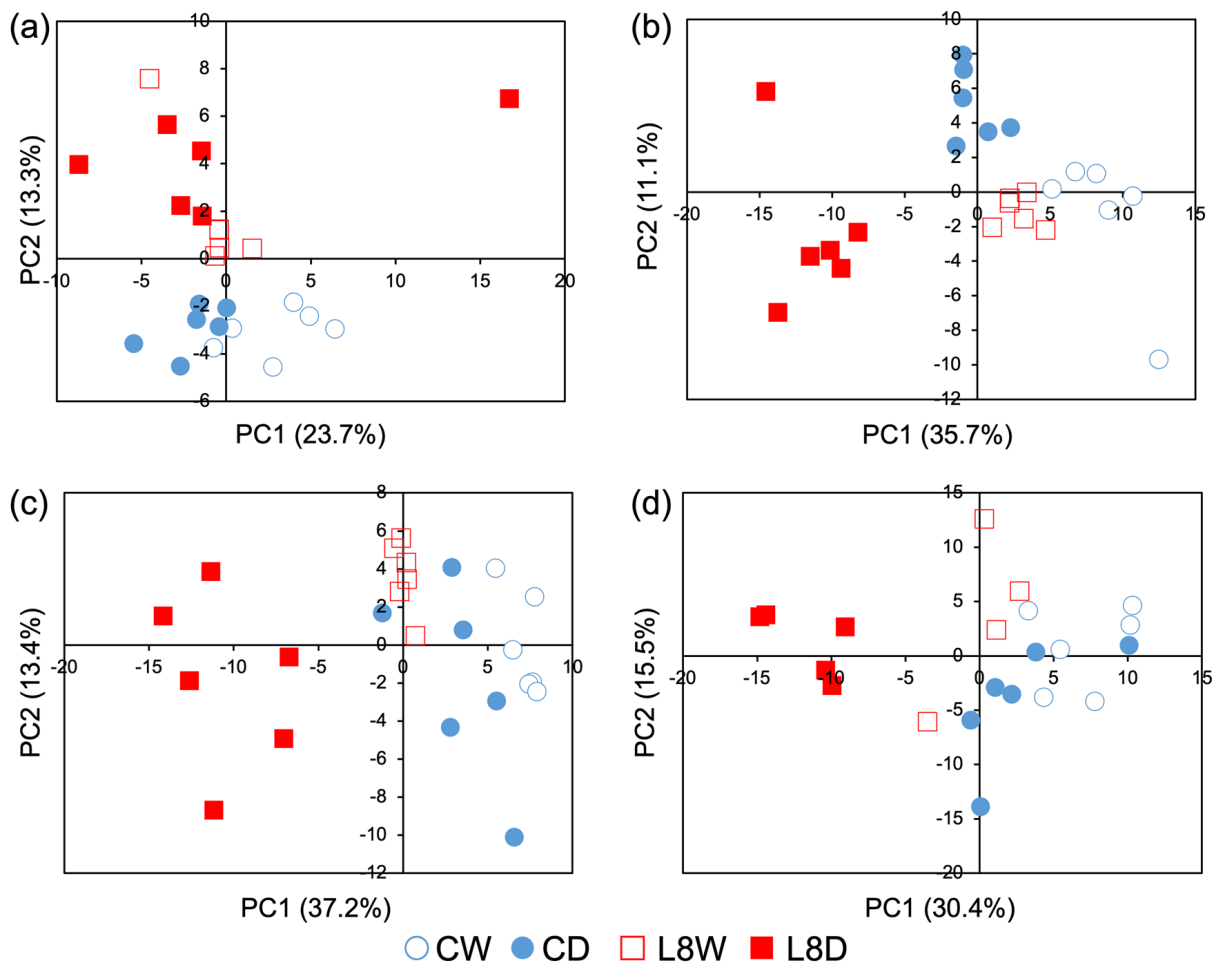
PCA of the metabolome. PCA score plots of metabolites in developing wheat seeds at days 2 (**a**), 5 (**b**), 7 (**c**), and 9 (**d**) were drawn using all detected metabolites. Samples were collected from different DR and WTR conditions according to their metabolite profiles.

Metabolomic analysis was performed using GC–MS to investigate metabolite accumulation patterns, and the same sample was used for transcriptomic analysis (Fig. S1). PCA was performed on each day analyzed to compare WTR and DR in lines C and L8 (C in WTR or DR, CW or CD; L8 in WTR or DR, L8W or L8D; Figs. 6 and S2). At day 0, genotypic differences were observed in C and L8. At day 2, all samples were distributed in similar regions, indicating that there were no effects of drought on metabolites in either line. However, genotypic differences were observed again. At day 5, drought-treated L8 samples were shifted furthest away from the other samples, indicating that some specific metabolites accumulate in L8 in response to drought. However, a slight change was also observed in CD. At days 7 and 9, the distribution tendency was similar to that at day 5, indicating that the metabolite accumulation pattern in L8 seeds was unaffected by progressive drought stress. However, although starch granule architecture and SSP fluctuations caused by drought stress were observed in line C, the metabolite accumulation pattern changed less than that of L8 at each day.

**Figure 7:**
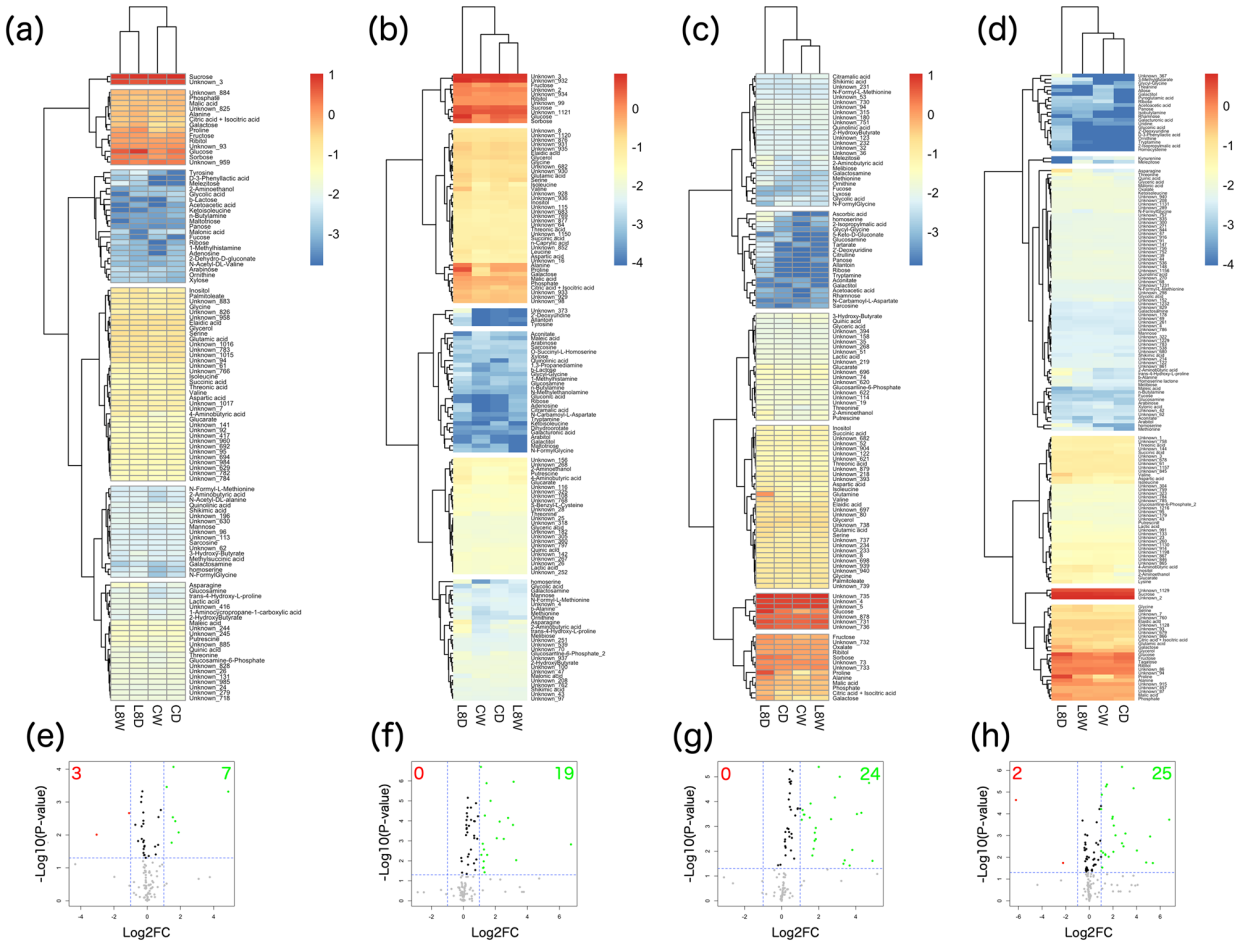
Hierarchical clustering and volcano plot analysis of the metabolome. Heatmaps of metabolites in developing wheat seeds at days 2 (**a**), 5 (**b**), 7 (**c**), and 9 (**d**) were drawn using all detected metabolites. The log_10_ transformation of the mean values of all detected metabolites was performed for hierarchical clustering. Volcano plots of metabolites in developing wheat seeds at days 2 (**a**), 5 (**b**), 7 (**c**), and 9 (**d**) were drawn to select differentially accumulated metabolites (DAMs). The threshold of significance was defined as P < 0.05 for downregulated (red dots, fold change ≤ 0.5) and upregulated metabolites (green dots, fold change ≥ 2.0).

Hierarchical clustering analyses were performed at days 0–9 (Fig. 7a–d). At day 2, the clades were separated by genotypes C and L8 rather than treatment. This suggests that at day 2, drought stress cannot change metabolite accumulation. At day 5, CD and L8W were in the same clade, suggesting that under mild drought stress, the control line has a similar status to TaPYLox, which has a transcriptome indicating mild drought stress, even under control conditions, because of its high ABA sensitivity^11^. At day 7, CW and L8W were in the same clade. CD gradually separated from CW and L8W, but L8D remained in a separate clade as at day 5. This suggests that in C, responses to drought stress would proceed apart from L8D affected by TaPYL overexpression. Intriguingly, at day 9, CD and CW were classified into the same clade. However, L8D belonged to an independent clade at days 5 to 9, unlike the other three samples.

Significantly up- and down- regulated metabolites (≥ twofold change and P < 0.05) were characterized by volcano plots under each drought conditions at days 2–9. Each day, CDvsCW, L8DvsCD, L8DvsL8W, and L8WvsCW were used to draw volcano plots (Figs. 7e–h and Figs. S3–6). As a result, there were 5, 7, 2, and 10 upregulated differentially accumulated metabolites (DAMs) of CDvsCW, L8DvsCD, L8DvsL8W, and L8WvsCW, respectively, at day 2. Downregulated DAMs were 1, 3, 1, and 0, respectively. At day 5, there were 10, 19, 17, and 13 upregulated DAMs of CDvsCW, L8DvsCD, L8DvsL8W, and L8WvsCW, respectively. Downregulated DAMs were 1, 0, 0, and 2, respectively. At day 7, there were 10, 24, 23, and 9 upregulated DAMs of CDvsCW, L8DvsCD, L8DvsL8W, and L8WvsCW, respectively. Downregulated DAMs were 0, 0, 1, and 1, respectively. At day 9, there were 3, 25, 12, and 8 upregulated DAMs of CDvsCW, L8DvsCD, L8DvsL8W, and L8WvsCW, respectively. Downregulated DAMs were 9, 2, 2, and 4, respectively. DAMs at days 2, 5, 7 and 9 are shown in Table S4.

There were many candidate pathways to explain the relationships between identified DEGs and DAMs. Pro and 4-hydroxy-proline determined as DAMs at day 9 were included in Arg and Pro biosynthesis in the KEGG pathway analysis (220 and 330, respectively). In this study, major seed components, SSP amounts, and starch granule architecture were investigated (Figs. 1 and 2). The amino acid compositions of SSPs were then investigated based on the CDS information of SSP genes stored in the EnsemblPlants database (https://plants.ensembl.org/Triticum_aestivum/Info/Index) (summarized in Tables 1 and S5). According to Table 1, Gln was the most common amino acid in all glutenin and gliadin proteins. Pro was the second most common amino acid found in almost all SSPs. The third most common one was serine (Ser) or leucine (Leu) in LMW-GSs and Leu in almost all gliadins. Interestingly, the first and second highest amino acid compositions of SSPs, including HMW- and LMW-GSs, and gliadin were nearly identical. This suggests that wheat SSPs may store specific amino acids for some purpose.

**Table 1:** Amino acid compositions of SSPs.

Gene_ID	Function	Amino acid compositions (%)							
		1st		2nd		3rd		4th	
TraesCS1B02G329711.1	HMW-glutenin	Q	25.9	G	14.4	P	10.3	S	8.5
TraesCS1B02G011700.1	LMW-glutenin	Q	36.9	P	12.8	S	8.1	L	7.8
TraesCS1B02G013500.1	LMW-glutenin	Q	32.6	P	14.6	L	8.6	S	7.4
TraesCS1D02G000200.1	LMW-glutenin	Q	31.6	P	12.7	L	8.8	S	7.2
TraesCS1D02G000300.1	LMW-glutenin	Q	33.3	P	13.8	S	8.8	L	8.5
TraesCS1D02G007400.1	LMW-glutenin	Q	29.2	P	11.1	L	8.4	S	8.4
TraesCS1D02G009400.1	LMW-glutenin	Q	34.3	P	13.4	S	8.6	L	6.9
TraesCS1D02G009900.1	LMW-glutenin	Q	31.7	P	10.9	S	9.6	L	7.9
TraesCS1D02G015100.1	LMW-glutenin	Q	32.1	P	15.1	L	8.5	S	7.4
TraesCSU02G149946.1	Gliadin	Q	35.5	P	14.1	L	7.9	I, S	5.2
TraesCSU02G149951.1	Gliadin	Q	36.5	P	13.9	L	7.8	I, S	5.1
TraesCSU02G153800.1	Gliadin	Q	31.6	P	15.3	L	7.8	I	5.8
TraesCS6A02G048900.1	Gliadin	Q	34.9	P	14.5	L	8.5	I	5.3
TraesCS6A02G049100.1	Gliadin	Q	31.1	P	14.1	L	8.8	I, S	5.3
TraesCS6A02G049200.1	Gliadin	Q	36.6	P	13.1	L	8.5	I	4.9
TraesCS6A02G049400.1	Gliadin	Q	31	P	14.8	L	9.2	I	5.3
TraesCS6A02G049600.1	Gliadin	Q	32.4	P	14.6	L	8.7	I	5.2
TraesCS6A02G049700.1	Gliadin	Q	35.4	P	14.1	L	8.1	I	5.4
TraesCS6A02G049800.1	Gliadin	Q	30.5	P	14.7	L	9.3	I	5.4
TraesCSU02G188800.1	Gliadin	Q	34.2	P	15.6	L	8.1	I, S	5.2
TraesCSU02G220200.1	Gliadin	Q	34	P	14.5	L	7.7	I, S	5.4
TraesCSU02G220600.1	Gliadin	Q	32.4	P	14.8	L	8.3	I, S	5.5
TraesCSU02G239000.1	Gliadin	Q	34.1	P	15.6	L	8.1	I, S	5.2
TraesCS6B02G065800.1	Gliadin	Q	37	P	13.6	L	8.5	S	5.4
TraesCS6B02G065856.1	Gliadin	Q	35.8	P	14.1	L	8.6	S	5.4
TraesCS6B02G066000.1	Gliadin	Q	34.3	P	13.9	L	8.6	I	5.4
TraesCS6B02G066100.1	Gliadin	Q	35.1	P	14.1	L	7.6	S	5.5
TraesCS6B02G086500.1	Gliadin	Q	37.4	P	13.9	L	7.4	I	5.5
TraesCSU02G108100.1	Gliadin	Q	32.6	P	14.4	L	8.2	I	5.2
TraesCSU02G108300.1	Gliadin	Q	34.7	P	14.3	L	8	I, S	5.3
TraesCSU02G108400.1	Gliadin	Q	32.8	P	15.7	L	8.2	I, S	5.1
TraesCSU02G108500.1	Gliadin	Q	31.1	P	15.7	L	8.4	I	5.6
TraesCSU02G108700.1	Gliadin	Q	31.2	P	15.2	L	7.1	S	6.4
TraesCS1D02G000700.1	Gliadin	Q	18.9	P	9	V	8	A, S	7.5
TraesCS1D02G001000.1	Gliadin	Q	31.2	P	14.8	L	6.7	I	6.44
TraesCS1D02G001200.1	Gliadin	Q	33.9	P	16.2	L	8	I	6.1
TraesCS1A02G007400.1	Gliadin	Q	30.2	P	16.1	L	6.7	I	5.6
TraesCS1A02G007405.1	Gliadin	Q	30.2	P	16.1	L	6.7	I	5.6
TraesCS1A02G007700.1	Gliadin	Q	19.5	P	9	V	8.5	A, T	7
TraesCS1B02G010400.1	Gliadin	Q	29.8	P	16.2	L	7	I	6
TraesCS1B02G010500.1	Gliadin	Q	29	P	16.5	L	7.4	I	6.1
TraesCS1B02G010600.1	Gliadin	Q	26.8	P	14.5	L	11.7	F, V	4.7
TraesCS1B02G010800.1	Gliadin	Q	33.9	P	16.7	L	7.6	I, S	5.6
TraesCS1B02G011000.1	Gliadin	Q	30.2	P	15.1	L	8.2	I	6.9
TraesCS1B02G011300.1	Gliadin	Q	18.7	P	7.9	S	7.9	V	7.9
TraesCSU02G272393.1	Gliadin	Q	34.2	P	25.9	F	9.5	L	5.1

Refer to the Table S5 caption if more information about Table 1 is required.

### Metabolite pathway map analysis using DEGs and DAMs

**Figure 8:**
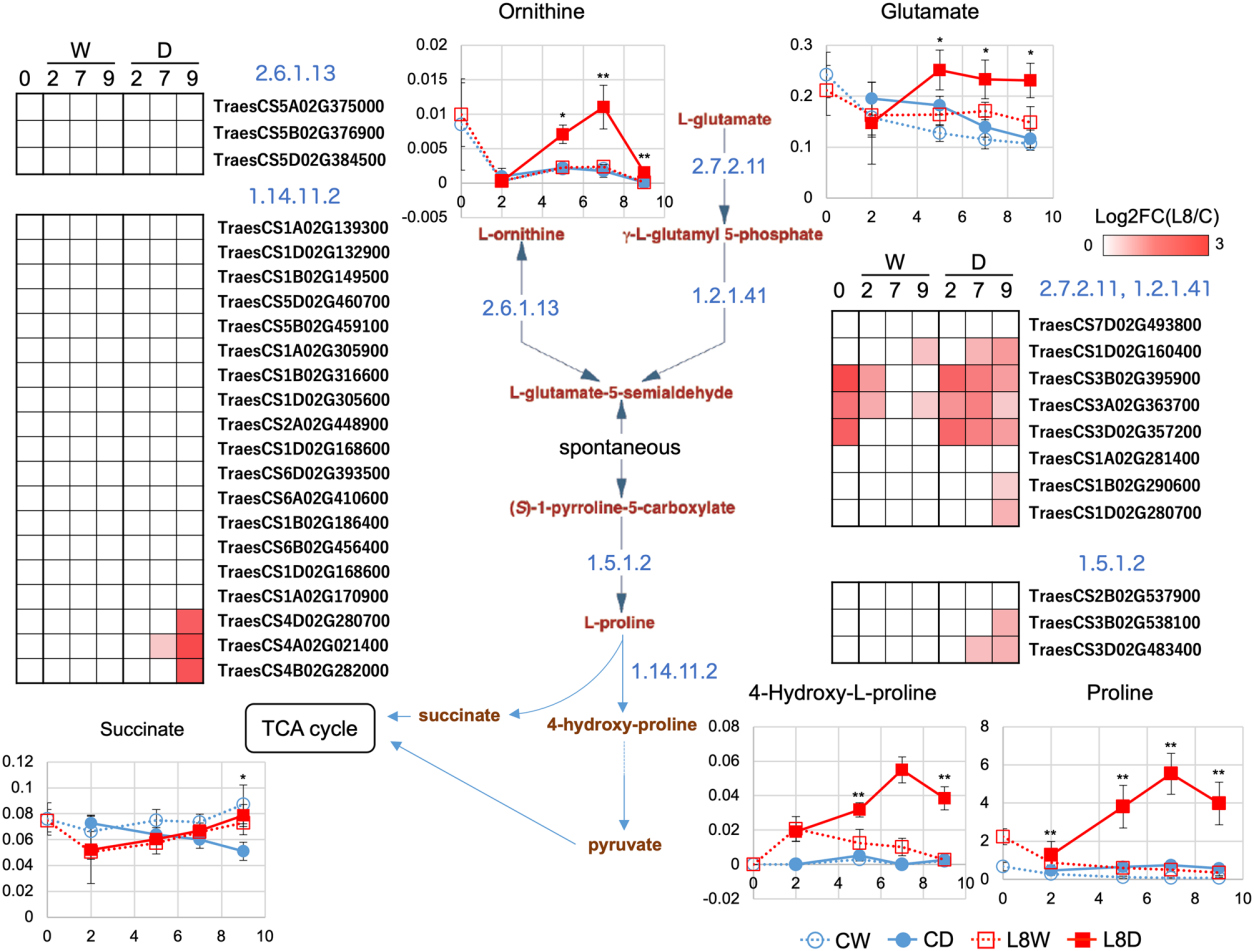
Pathway map analysis of Pro biosynthesis. Differentially expressed genes (DEGs) and differentially accumulated metabolites (DAMs) were used to draw the pathway map. Orn and Glu were used as initial metabolites, and the downstream pathway was drawn to succinate. DEGs were shown by color panels, and DAMs were indicated by line graphs. Enzymatic reactions are indicated with blue-colored EC numbers. ** and * indicate DAMs (L8DvsCD) and metabolites (fold change < 2, P < 0.05 in L8DvsCD) in the line graph panels, respectively. In the line graphs, values of the x- and y-axes are durations after treatment (Fig. S1) and normalized values calculated by AIoutput, respectively.

**Figure 9:**
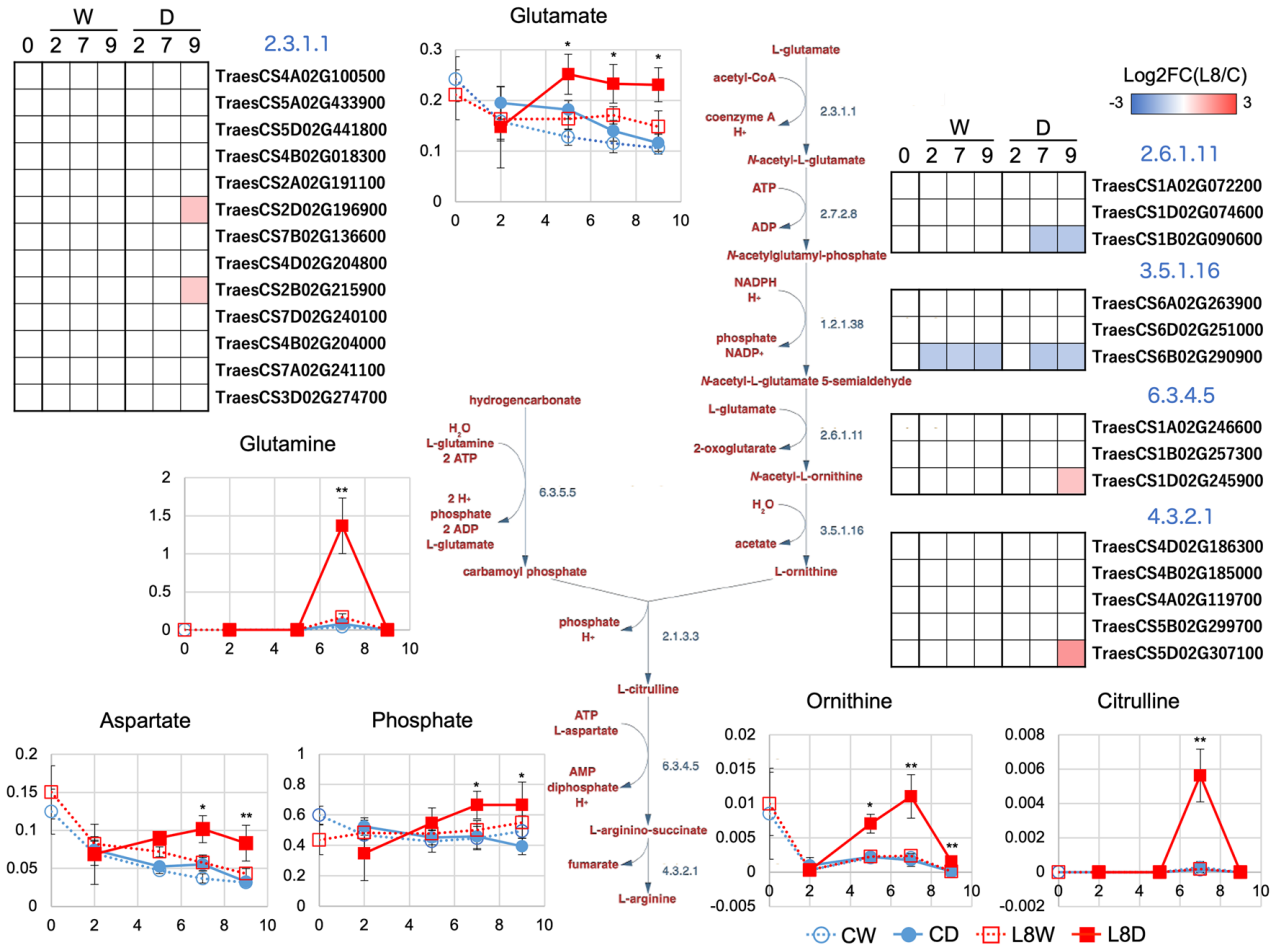
Pathway map analysis of Arg biosynthesis. DEGs and DAMs were used to draw the pathway map. Glu was used as the initial metabolite, and the downstream pathway was drawn to Arg. DEGs were shown by color panels, and DAMs were indicated by line graphs. Enzymatic reactions are indicated with blue-colored EC numbers. ** and * indicate DAMs (L8DvsCD) and the metabolites (fold change < 2, P < 0.05 in L8DvsCD) in the line graph panels, respectively. In the line graphs, values of the x- and y-axes are durations after treatment (Fig. S1) and normalized values calculated by AIoutput, respectively.

A metabolite pathway map was constructed based on DEGs and DAMs and on the amino acid compositions described above (Figs. 8 and 9). The pathway maps were based on the Plant Metabolic Pathway (https://plantcyc.org) and KEGG pathway (https://www.kegg.jp/kegg/pathway.html) databases. In Fig. 8, Pro is biosynthesized from glutamate (Glu) and ornithine (Orn) via two or three enzymatic reaction steps. No genes for enzymes that catabolize Orn were found as DEGs. However, many genes for enzymes that catabolize Glu were upregulated under drought conditions in L8. In addition, Pro biosynthetic genes were upregulated in L8 under severe drought. This suggests that Pro biosynthesis in response to drought is activated through Glu rather than Orn in L8. Biosynthesized Pro would be utilized effectively to maintain SSPs in L8 under drought conditions. Furthermore, 4-hydroxy-proline and succinate also increased following Pro accumulation in L8D but not in CD. This suggests that a mechanism for preventing Pro overaccumulation functions and can contribute to activation of the downstream TCA cycle. In Fig. 9, Arg is shown to be synthesized through eight or four enzymatic reaction steps when the initial compound is Glu or hydrogen carbonate, respectively. In this pathway map, two DEGs involved in enzymatic reactions starting from Glu were downregulated. However, Orn accumulated.

## Discussion

This study used metabolomic and transcriptomic data to comprehensively indicate how drought stress affects grain development. According to phenotypic data (Figs. 1 and 2), seed shrinkage by drought stress was likely due to insufficient starch granule growth. Meanwhile, it was unlikely that SSPs affected seed shrinkage. This difference suggests that SSPs’ biosynthesis may be protected but not starch biosynthesis. The other study indicated that fewer and smaller starch granules A and B were observed under drought stress like our findings^19^. According to the paper, the proteomic data also showed inhibition of photosynthesis-related pathway and starch biosynthesis similarly to our transcriptomic data.

Cultivation under drought stress conditions reduced glutenin amounts in mature seeds (Fig. 2). Transcriptomic GO enrichment and KEGG pathway analysis indicated that translation (GO:0006412) and aminoacyl-tRNA biosynthesis (970) changed significantly under drought conditions (Figs. 4 and 5). There are no categories for SSPs, even in the GO slim database and the wheat genome database of EnsemblPlants. Thus, GO enrichment analysis could not find GO terms for SSPs. Because the gene IDs of SSPs were also only partially annotated in the database, gene IDs were collected from Gao et al.^18^ and summarized using the fold-change values of DEGs in Table S3. Consequently, no HMW- and only one LMW-GS were detected as DEGs under drought conditions at days 7 and 9 when comparing L8 and C. This suggests that glutenin amounts are independent of transcript accumulation. According to GO enrichment and KEGG pathway analysis, protein biosynthesis steps could greatly contribute to glutenin accumulation (Fig. 2). However, 23 gliadin genes were upregulated in L8 at day 9 under drought conditions (Table S3). Particularly, 13 out of 14 omega-gliadins were increased in L8. This suggests that gliadin gene expression may be strongly affected by severe drought stress in CD.

KEGG pathway analysis also showed that Pro and Arg biosynthesis (220 and 330, respectively) was upregulated in L8 under drought conditions (Figs. 4, 5). Particularly, Pro is a main component of SSPs (Table 1). In addition, Pro is well known as a compatible solute induced by high salinity, high light, UV irradiation, heavy metals, oxidative stress, various biotic stresses, and drought^20^. Pro is accumulated to high levels in drought-tolerant rice varieties^21^. The relationship between Pro and salt- or freezing-tolerance has been reported in Arabidopsis^22,23^. However, it remains unclear why Pro accumulates as a compatible solute in plants damaged by these stresses^20^. This study is the first time that metabolite profiling was performed in wheat spikelets during seed development. Wheat has many specific SSPs that determine end-product flour quality in addition to those shared with rice and Arabidopsis. Pro was shown to be a major component of SSPs in this study (Tables 1 and S5). Thus, our findings determined a reason for Pro accumulation by understanding SSPs as determinants of wheat seed quality. Accumulating Pro is important for maintaining SSPs inside seeds and may contribute to nutrition storage for the next generation.

KEGG pathway analysis also showed that starch metabolism-related pathways (500, 52, and 10) were involved in wheat seed development during drought. According to Table S3, nine genes (four sucrose synthases, four 1,4-alpha-glucan branching enzymes, and one debranching enzyme) were upregulated in L8 under drought conditions at day 9. Starch is biosynthesized through amylose straight-chain elongation and amylopectin branched modification^24^. According to the upregulated genes discussed above, 1,4-alpha-glucan branching enzymes essentially function to form amylopectin. Starch granules are complexes consisting of long and multi-branched amylopectin. Downregulation of branching enzymes in CD is likely to inhibit starch granule formation. This suggests that the starch-biosynthesis step affected by progressive drought stress could be the branching step of amylose. Sucrose synthase was also downregulated in CD, and a pathway map analysis of sugar around sucrose was constructed (Fig. S7). First, sucrose was not detected as a DAM every time in Table S4. Second, there were no significant differences between CD and L8D according to Fig. S7. This was not consistent with the transcriptomic data (Table S3). Metabolomic measurements using GC–MS were not able to quantify sucrose. To validate this inconsistency, sucrose should be measured using a more quantitative method, such as HPLC using an electrochemical detector.

Puroindoline genes are listed (Table S3) using information shown in Kiseleva et al.^25^. Several of these genes were also upregulated in L8 at day 9. This suggests that there might be some negative effects of drought stress on puroindoline gene expression in CD. However, an additional experiment is required to determine the effect of drought on grain texture, softness, or hardness in both lines.

Ingredients of SSPs and starch are limited to amino acids and sugars, respectively. These compounds may be selected as functionally optimal storage components because they are compatible compounds. The storage of these compounds in seeds may mean they are accumulated rapidly to protect tissues when plants are exposed to stress conditions. They may also function to support plant growth in the next generation. This function is known as the “transgenerational effect”^26–28^. Though there is epigenetic and transcriptional evidence for this effect, metabolic evidence has not been reported yet. Pro and sugars stored in SSPs and starch, respectively, are utilized not only as nutrition but as compatible compounds in generated seeds. Thus, this accumulation of compatible compounds might contribute to the transgenerational effect. Indeed, wheat seeds with increased Pro caused by terminal drought stress acquire more drought tolerance^17^. This provides support for the possibility that these metabolites function transgenerationally to adapt to future environments for progeny.

Finally, *TaPYL4* overexpression did not have strong effects on the seed phenotypes under WTR (Figs. 1 and 2) in this and the previous study^11^. However, there are several slight differences between TaPYLox and C such as starch granule constitution, heading date, plant height, productive tiller number. Likewise, these phenotypes are reported in overexpressed wheat plants of *TaPYL1-1B* (*TraesCS1B02G206600*)^29^. In rice multiple *PYL*s mutant line, they are contradictory^30^. This suggests that *TaPYL4* and the other homeologs in wheat could be useful as target genes for drought-tolerant crop breeding without end-product penalties. However, because slight transcriptomic and metabolomic changes were observed related to *TaPYL4* and its homeologs, altering their expression could affect plant growth and vulnerability to other stresses in an agricultural environment. Therefore, we should use the TaPYLox wheat to verify this possibility by a field trial.

## Methods

### Plant materials and progressive drought treatments

Spring wheat (*Triticum aestivum* L. cv. Fielder; Accession number, KT020-061) was provided by the National BioResource Project–Wheat, Japan. Using this cultivar, the transgenic wheat overexpressing the ABA receptor gene *TaPYL4* (TaPYLox) was generated in our previous study^11^. Though multiple TaPYLox lines were obtained, one TaPYLox lines (L8) and its null segregant, as the control line (C), were used in all experiments of this study. Experimental research on cultivated varieties complied with relevant institutional, national, and international guidelines and legislation. The seeds were stratified at 4 °C for 4 days and cultivated for 7 days in pots (ϕ 7.5 cm × D 6.5 cm) containing commercial garden soil (Oishii Yasai Wo Sodateru Tsuchi, CAINZ, Saitama, Japan). They were cultivated in rectangular pots (internal dimension = L 30 cm × W 18 cm × H 18 cm) with water containing 10 mL/L of liquid fertilizer (N P K 6. 10. 5, HYPONex, Osaka, Japan) until three or four spikes flowered (approximately 7 days post-anthesis) under well-watered conditions^31^. The plants were grown in a climate-controlled growth chamber (Espec, Osaka, Japan). The chamber was maintained at 25 °C (14-h light)/15 °C (10-h dark), with relative humidity levels of 40% (light)/60% (dark). The water supply was stopped 7 days after anthesis for drought-treated samples (DR days 2, 5, 7, and 9) and continued for the well-watered ones (WTR days 2, 5, 7, and 9). Soil water potential (SWP, kPa) values at each point are shown in Fig. S8. SWP was calculated based on measured soil water content values (%) using the calibration equation we proposed previously^11^.

### Observation of horizontal sections of seeds

Mature seeds of the C, L8, and L17 lines obtained in our previous study were used for scanning electron microscope (SEM) analysis. The seeds were cut horizontally using a razor blade. Their horizontal surfaces were observed with a Quanta250 SEM (Thermo Fisher Scientific, Waltham, MA, USA). To measure the sizes of starch granules using Image J, SEM observation was performed at a magnification of 1600 × .

### Seed storage protein analysis

The same series of seeds as for SEM analysis was used for seed storage protein analysis. Three technical replicates were performed for each of four biological replicates. Three fully developed kernels were selected from each plant grown under each condition. The glutenins extracted from the powder of three kernels were separated by SDS–PAGE according to the methods of Tanaka et al.^15^^,^^32^. Glutenin bands were stained with colloidal Coomassie Brilliant Blue G-250 solution according to the method of Dyballa and Metzger^33^. The stained gels were scanned using a flatbed scanner, and the protein band patterns were visualized. To measure protein quantity, the band densities were normalized by loading the same amount of sample extracted from the bread wheat cultivar ‘Chinese Spring’ onto each gel and then analyzed using JustTLC software Version 4.5 (Sweday).

### Transcriptome analysis

The transcriptome was analyzed in six biological replicates of RNA samples. Frozen powder prepared for the metabolomic analysis was used to extract total RNA using TRIzol (Thermo Fisher Scientific). The mRNA extraction and the sequencing library preparation was performed using the NEXTflex Rapid DIR mRNA Bundle (PerkinElmer, Waltham, MA, USA). Sequencing on a NovaSeq 6000 system generated an average of 53.8 million paired-end reads (2 × 151 nucleotides) per library. Data analysis was performed as described in our previous report^11^. Briefly, manually qualified sequencing reads were mapped to the cDNAs of the wheat reference genome, IWGSC RefSeq v1.0^34^; digital expression values for each transcript and differential expression were analyzed by utilizing the kallisto–sleuth pipeline, version 0.44.0^35^. Gene ontology (GO) analysis was performed utilizing GOstats^36^, version 2.46.0 in R^37^. The GO slim accessions for wheat genes were collected from the Ensembl Plants database (release-49, https://plants.ensembl.org/).

### Metabolomic analysis

Spikelets at each stage were collected and flash-frozen in liquid nitrogen. Then these were pulverized using a Multi-Beads Shocker (Yasui Kikai, Osaka, Japan) and lyophilized using a VD-550R freeze-dryer (TITEC, Saitama, Japan). The freeze-dried powder (2 mg) was used to prepare samples for metabolomic analysis. The powder was added to 80% methanol, and 10 mg of ribitol was added as an internal standard for retention-time correction. The resuspended solution was then incubated at 25 °C for 24 h in the dark after vortexing for 5 min. MilliQ water and chloroform were added, and the mixture was vortexed and centrifuged for 5 min. The supernatant was collected and dried using a vacuum evaporator. The dried sample was then derivatized for GC–MS measurements. The dried pellet was added to 20 mg/ml methoxyamine hydrochloride (Sigma-Aldrich, St. Louis, MO, USA) in pyridine (Wako, Osaka, Japan) and mixed at 30 °C for 2 h. The solution was added to BSTFA-TMCS (TIC, Tokyo, Japan) and mixed at 37 °C for 30 min.

The derivatized solution was loaded into a GC–MS system (GCMS-TQ8040 NX; Shimadzu, Kyoto, Japan) equipped with a DB-5MS column (30-m long, 0.25-mm internal diameter, 0.25-µm film thickness; Agilent Technologies, Santa Clara, CA, USA) with a 1:20 split ratio. Separations were performed using the following temperature gradient: 80 °C, 2 min; 80 °C–330 °C, 15 °C/min; and 330 °C, 6 min. The injector temperature was maintained at 230 °C, and the ion source and interface temperature were 200 °C and 250 °C, respectively.

The obtained chromatographic data were scaled using MetAlign (http://www.metalign.wur.nl/UK/Download+and+publications/)^38,39^. The detected peaks were identified using AIoutput (http://prime.psc.riken.jp/compms/others/main.html#AIoutput)^40^.

### Supplementary Information

Supplementary Figures.

Supplementary Legends.

Supplementary Table S1.

Supplementary Table S2.

Supplementary Table S3.

Supplementary Table S4.

Supplementary Table S5.

## Data Availability

The raw and processed data of mRNA-seq analysis were deposited in the NCBI Gene Expression Omnibus (GEO) database under a specific accession number (GSE227374; secure token, qbmdsqaapheplkz). In-house codes applied in this study are available through a GitHub deposit (https://github.org/junesk9).
