# Supplementary figures and images for: Metabolic and transcriptomic profiling during wheat seed development under progressive drought conditions
Source: Sci Rep. 2023 Sep 11;13:15001. doi: 10.1038/s41598-023-42093-2 (PMC10495411; doi:10.1038/s41598-023-42093-2)

Fig.S1

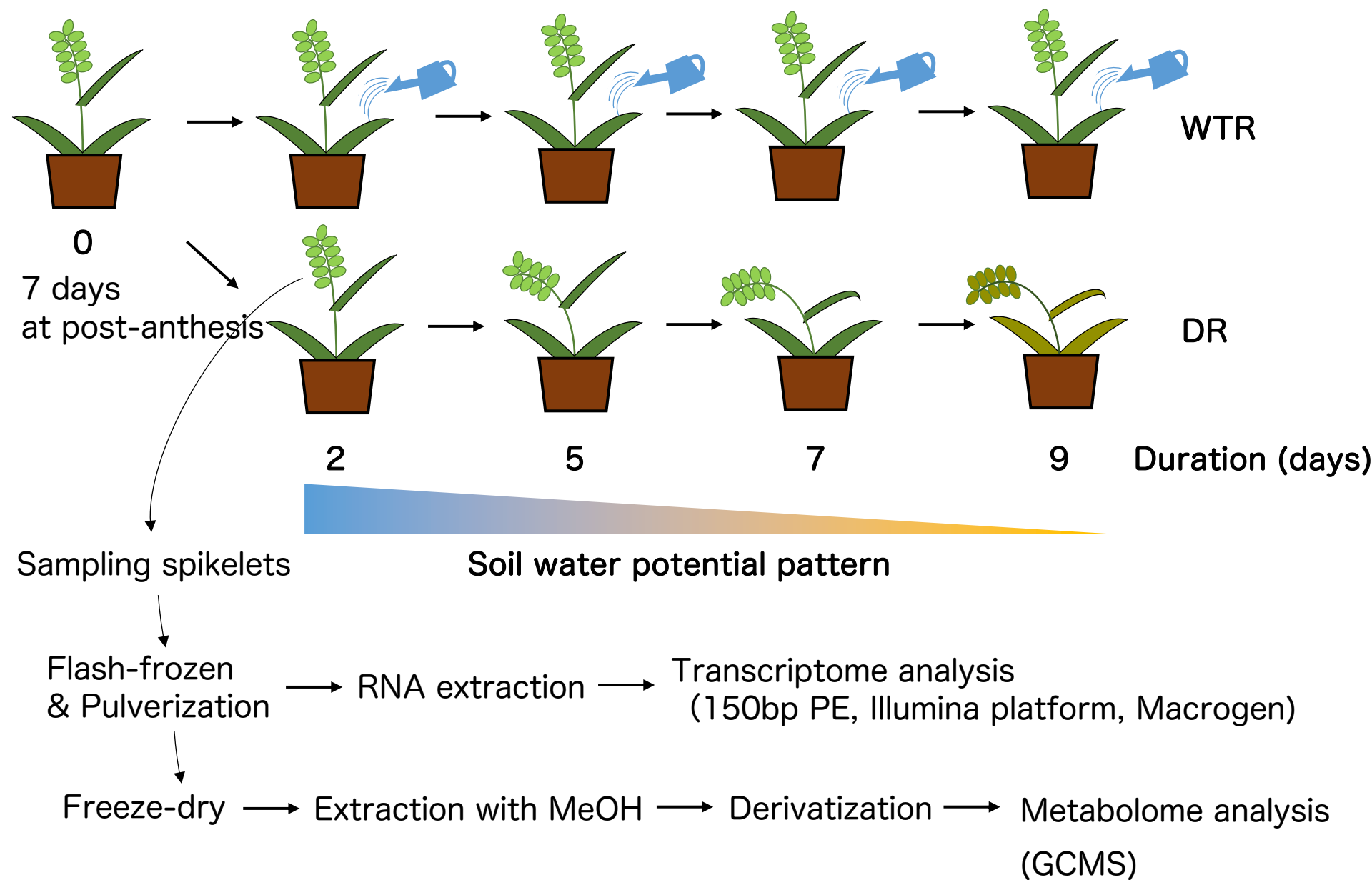

Fig.S2

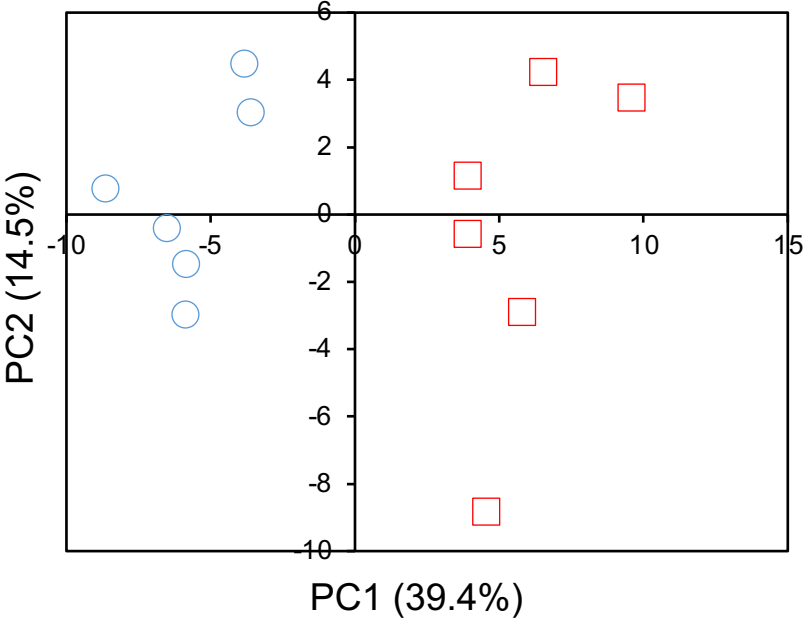

Fig.S3

CD vs CW

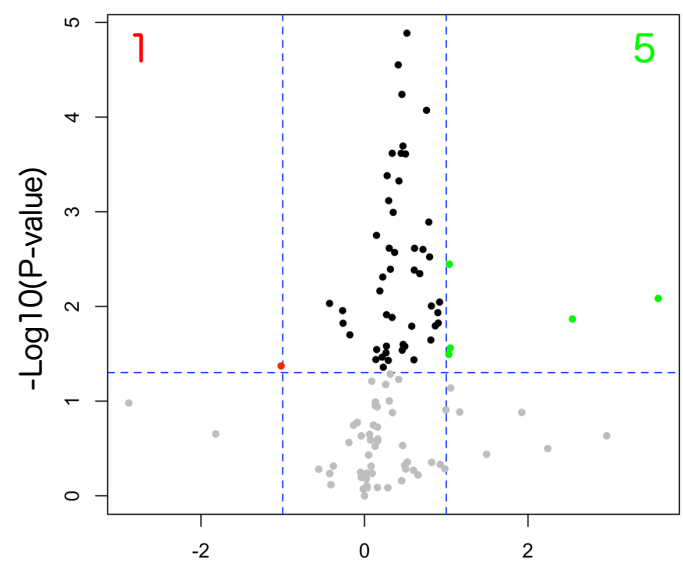

L8D vs L8W

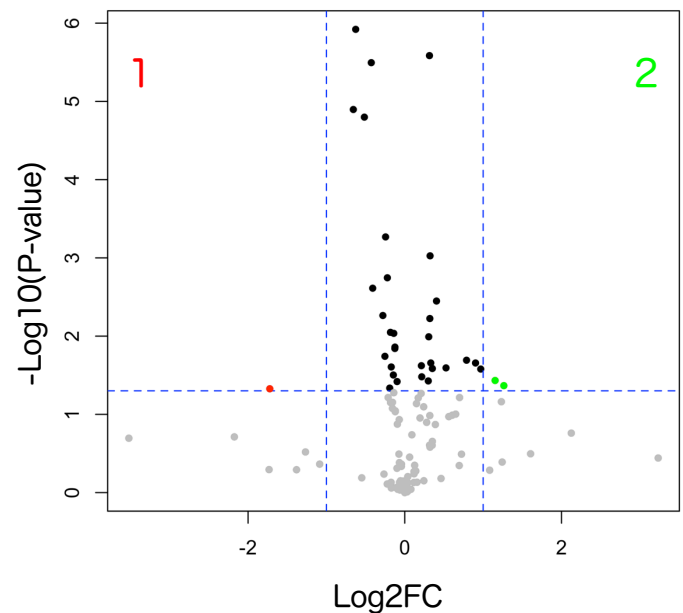

L8W vs CW

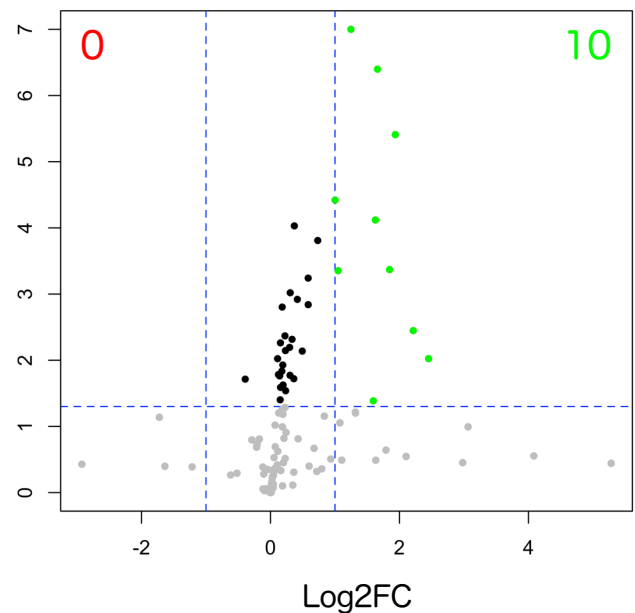

Fig.S4

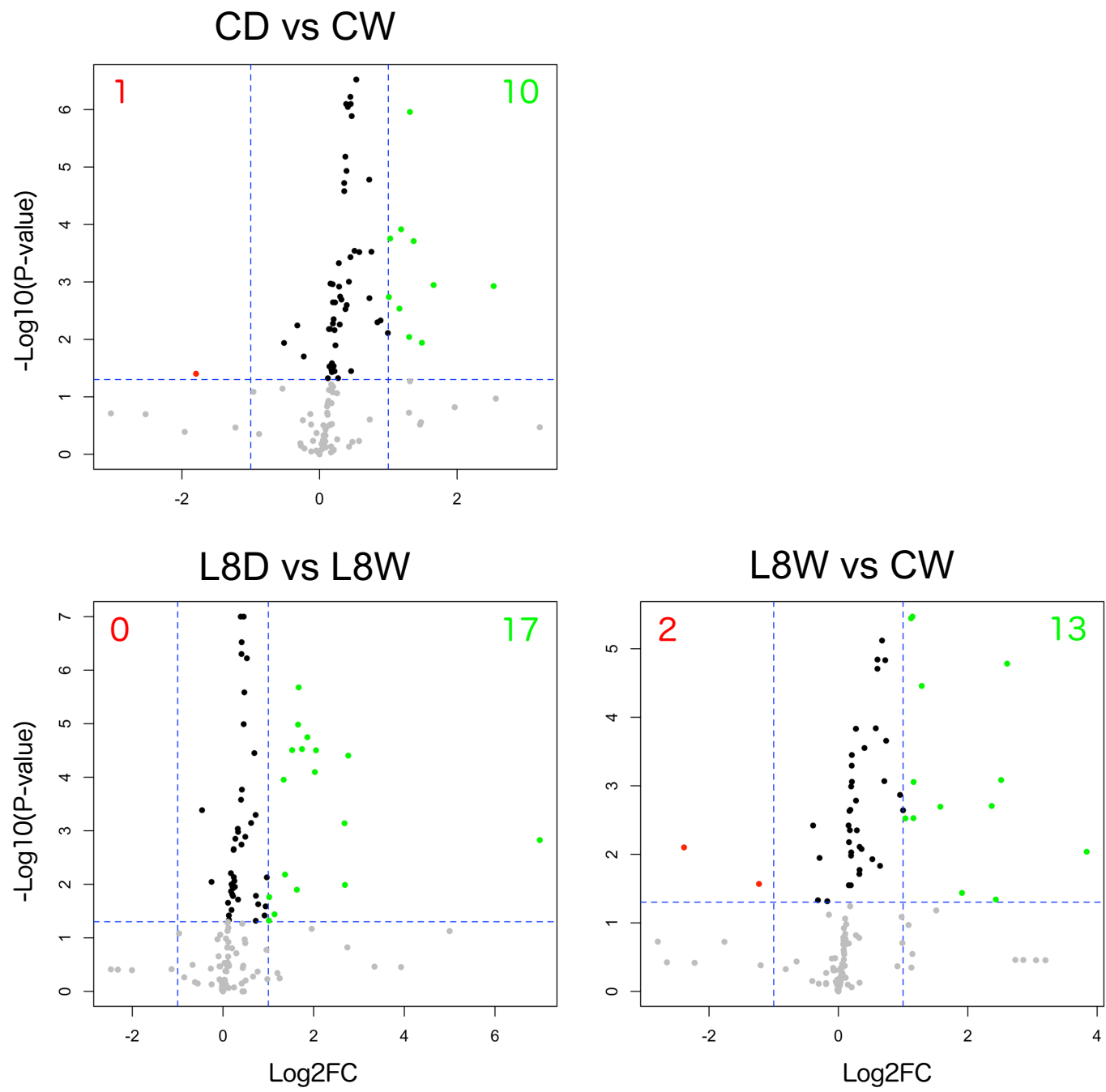

Fig.S5

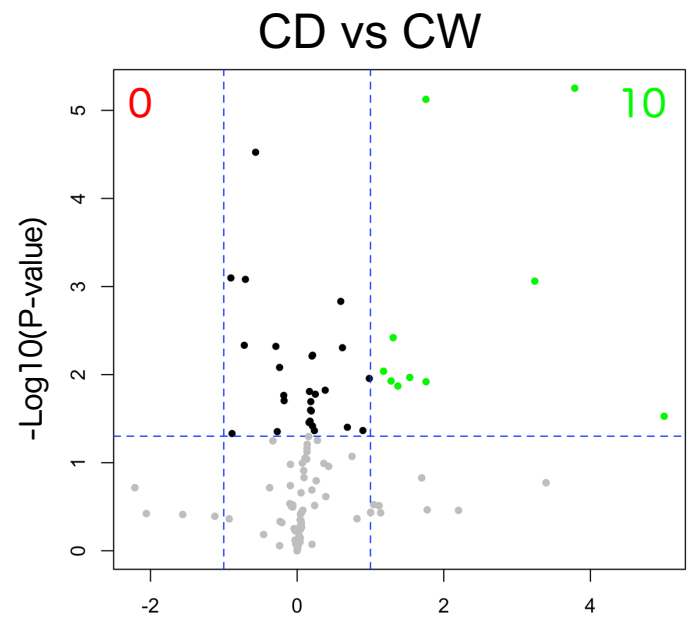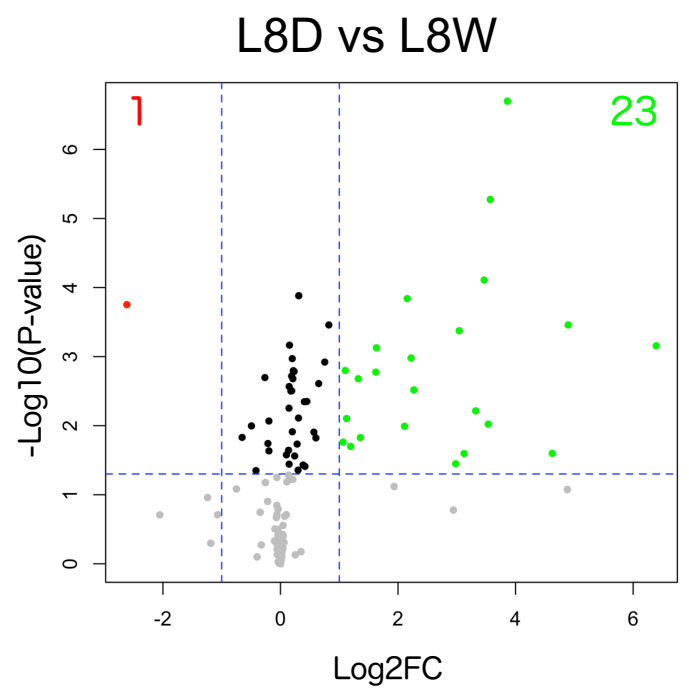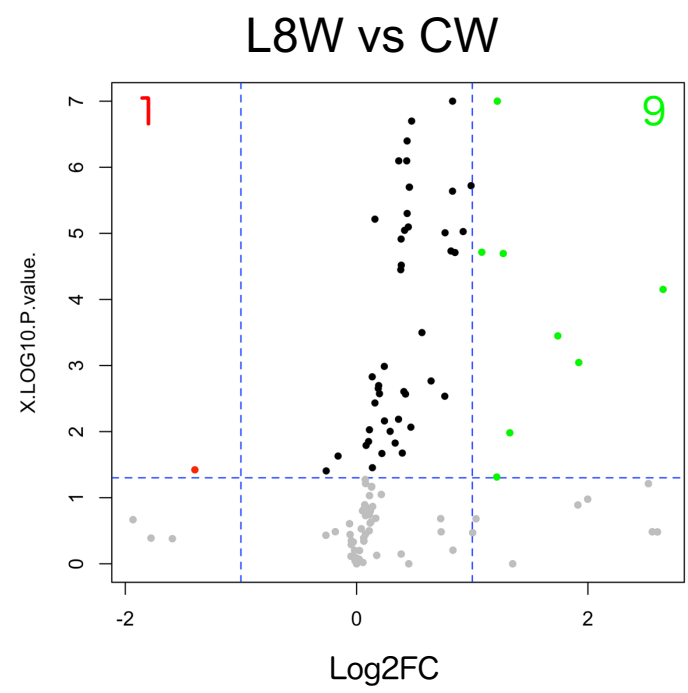

Fig.S6

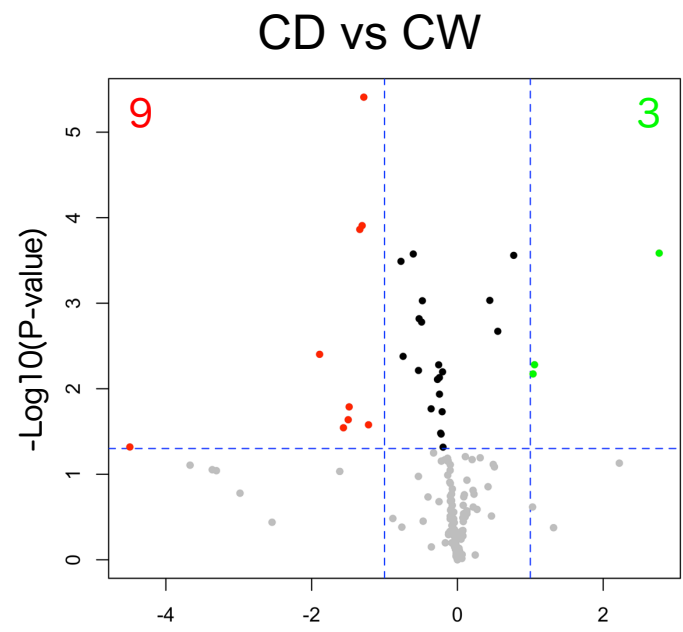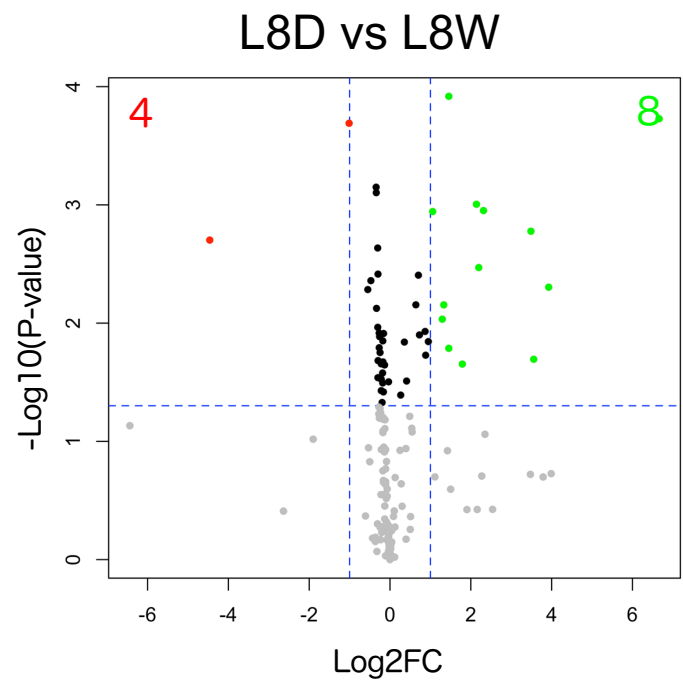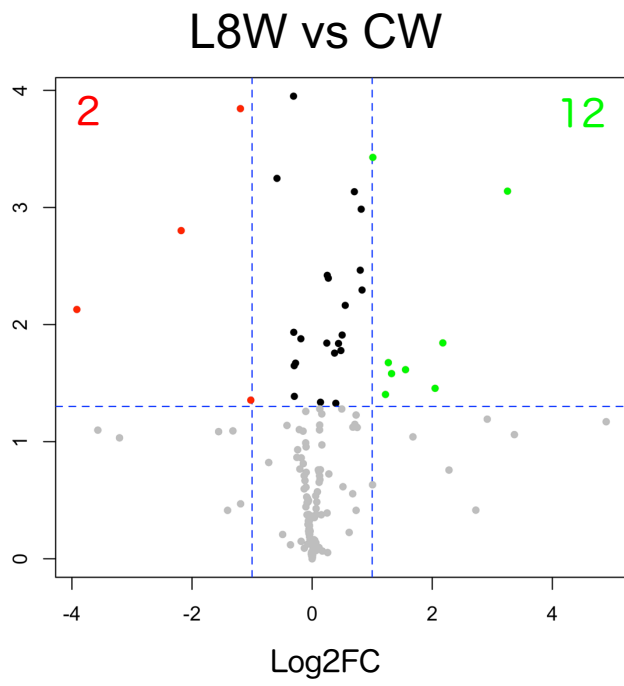

Fig.S7

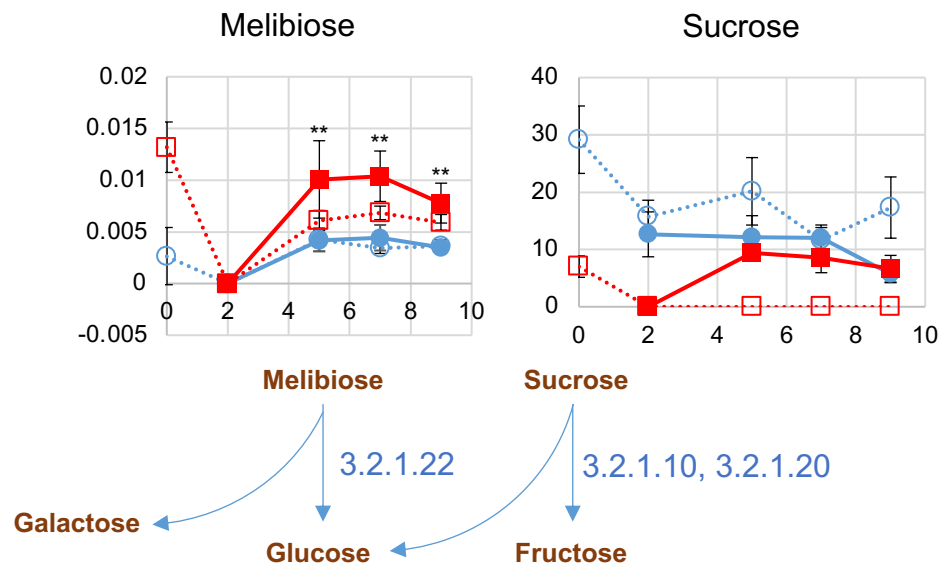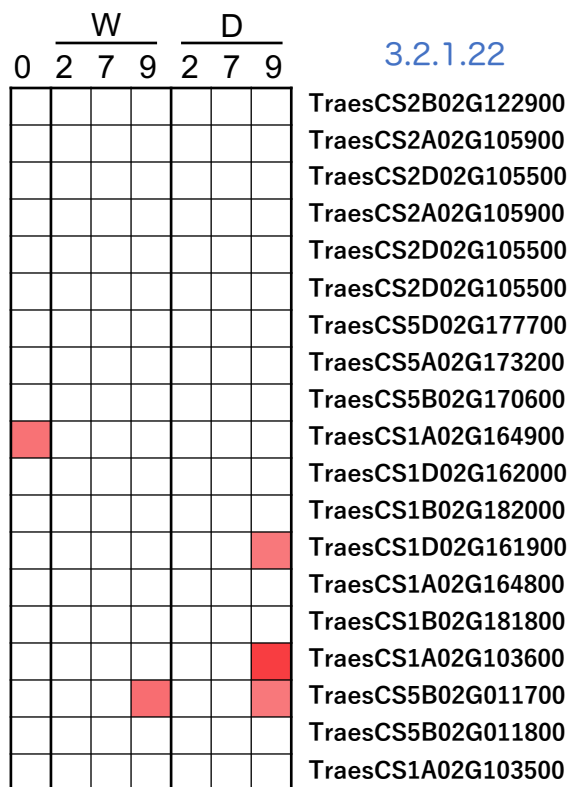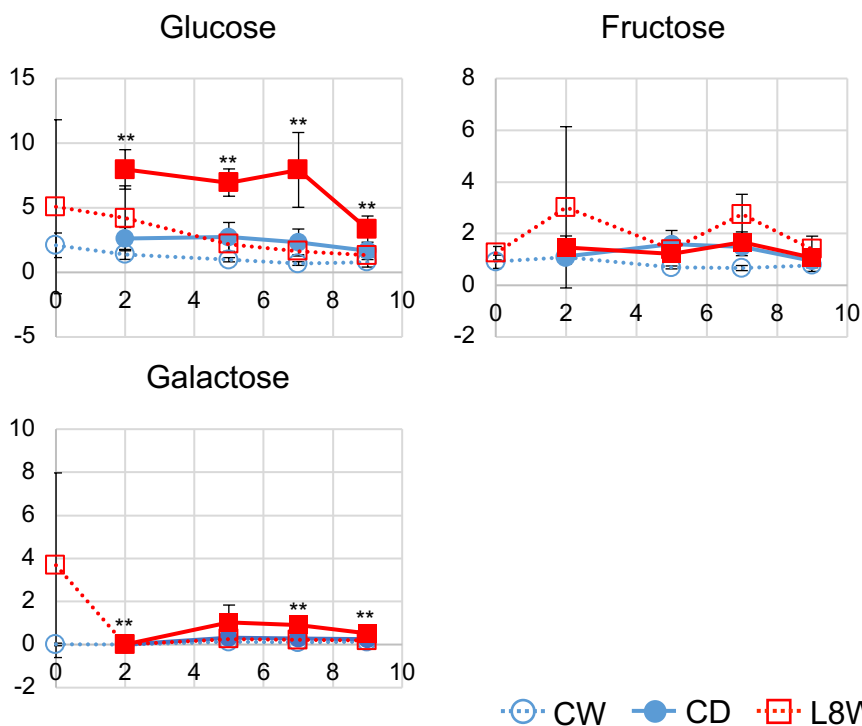

Fig.S8

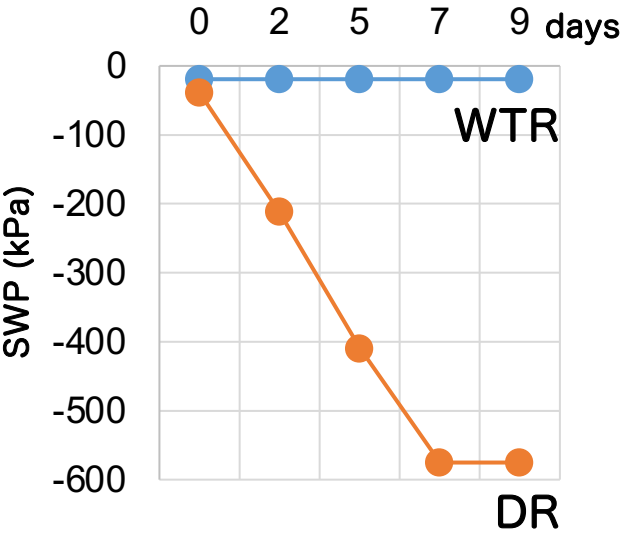

Fig.S9

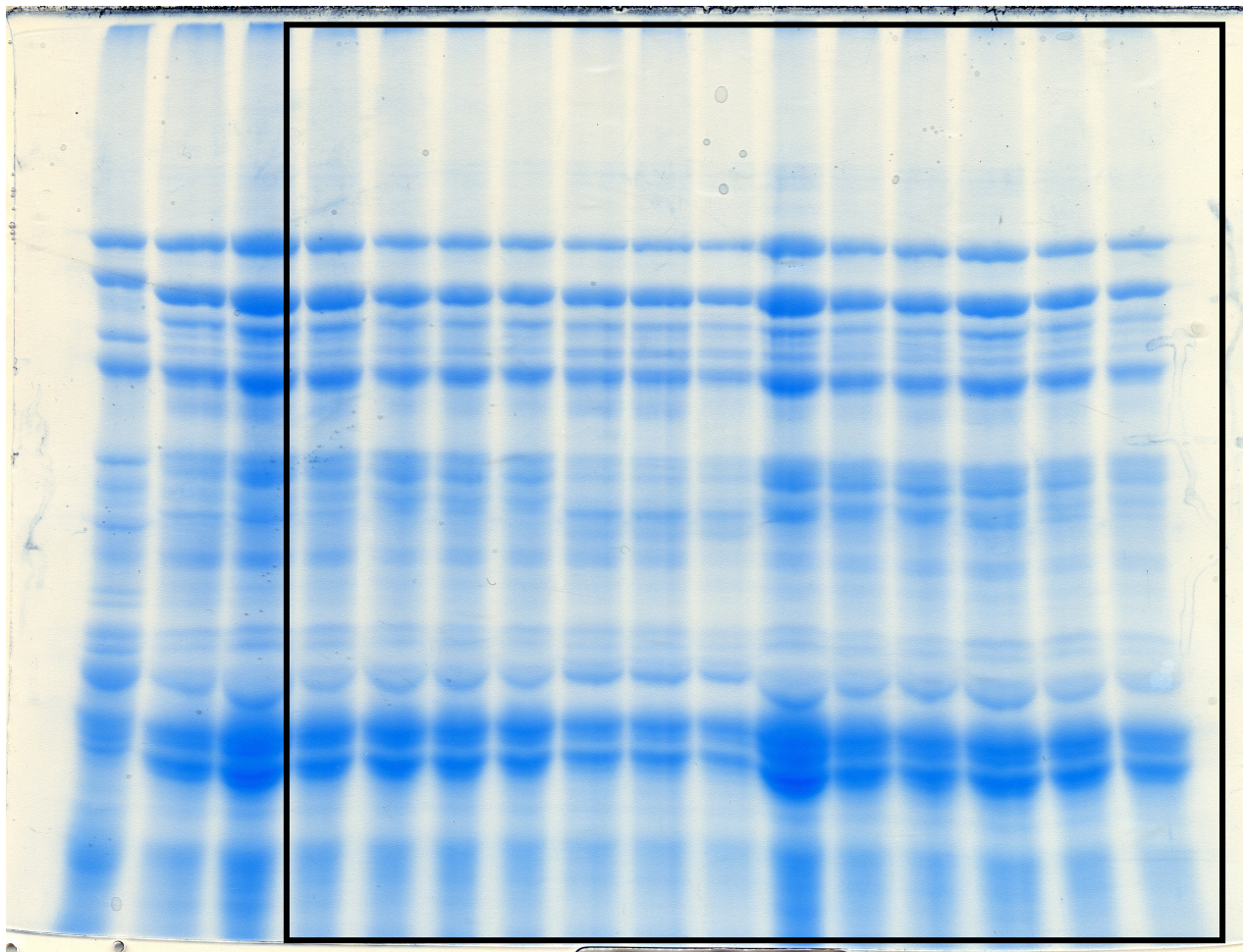

Trimmed for main Fig.2

Supplement: Supplementary file 1 — Supplementary Figures. [file 41598_2023_42093_MOESM1_ESM.pdf]
