# Supplementary material for: Metabolic and transcriptomic profiling during wheat seed development under progressive drought conditions
Source: Sci Rep. 2023 Sep 11;13:15001. doi: 10.1038/s41598-023-42093-2 (PMC10495411; doi:10.1038/s41598-023-42093-2)
Supplement: Supplementary file 2 — Supplementary Legends. [file 41598_2023_42093_MOESM2_ESM.docx]

Fig. S1. The design of this study.

Fig. S2. PCA of the metabolome at day 0.

Fig. S3. Volcano plots of the metabolome analysis at day 2 (CDvsCW, L8DvsL8W and L8WvsCW).

Fig. S4. Volcano plots of the metabolome analysis at day 5 (CDvsCW, L8DvsL8W and L8WvsCW).

Fig. S5. Volcano plots of the metabolome analysis at day 7 (CDvsCW, L8DvsL8W and L8WvsCW).

Fig. S6. Volcano plots of the metabolome analysis at day 9 (CDvsCW, L8DvsL8W and L8WvsCW).

Fig. S7. Pathway map analysis of sugar biosynthesis.

Fig. S8. Water decremental pattern of SWP after withdrawing.

Fig. S9. The full-size gel photo of Fig.2a.

Table S1. KEGG and GO terms shown in Fig.3.

Table S2. The percentages of DEGs belonging to carbohydrate metabolic process (GO:0005975) or protein metabolic process (GO:0019538) within all upregulated ones.

Table S3. All SSPs, Starch biosynthesis-related and puroindline genes annotated in EnsemblPlants.

The numbers in Before, DR2, 7 and 9 indicate fold changes of DEGs compared between L8 and C. “#N/A” means not DEGs.

Table S4. DAMs detected from volcano plots at day 2, 5, 7 and 9.

Table S5. Amino acid composition of all SSPs in Table S2.
